# Reading on Paper and Screen among Senior Adults: Cognitive Map and Technophobia

**DOI:** 10.3389/fpsyg.2017.02225

**Published:** 2017-12-19

**Authors:** Jinghui Hou, Yijie Wu, Erin Harrell

**Affiliations:** ^1^School of Communication, Florida State University, Tallahassee, FL, United States; ^2^Department of Psychology, Florida State University, Tallahassee, FL, United States

**Keywords:** older adults, reading, mobile technology, cognitive map, technophobia

## Abstract

While the senior population has been increasingly engaged with reading on mobile technologies, research that specifically documents the impact of technologies on reading for this age group has still been lacking. The present study investigated how different reading media (screen versus paper) might result in different reading outcomes among older adults due to both cognitive and psychological factors. Using a laboratory experiment with 81participants aged 57 to 85, our results supported past research and showed the influence of cognitive map formation on readers’ feelings of fatigue. We contributed empirical evidence to the contention that reading on a screen could match that of reading from paper if the presentation of the text on screen resemble that of the print. Our findings also suggested that individual levels of technophobia was an important barrier to older adults’ effective use of mobile technologies for reading. In the *post hoc* analyses, we further showed that technophobia was correlated with technology experience, certain personality traits, and age. The present study highlights the importance of providing tailored support that helps older adults overcome psychological obstacles in using technologies.

## Introduction

The senior population has been increasingly integrating technology into their everyday lives. One area of technology that older adults particularly embrace is electronic books. Known as the most avid book consumers, older adults also make up the highest percentage of tablet and e-reader owners according to a national poll (Harrison Interactive, 2012). Reading has proven to bring great benefits to older adults, including providing mental stimulation to the brain, slowing memory decline, reducing stress and enhancing sleep, and possibly delaying the onset of dementia and Alzheimer’s disease (Kawashima et al., 2005; Uchida and Kawashima, 2008; Nouchi et al., 2012; Kulason et al., 2016). Digital mobile devices may be particularly useful for senior readers because their advantageous technology features, such as backlit displays, adjustable text sizes, portable and lightweight frame, and touchscreens with large buttons, could accommodate for many common age-related limitations (Jochems et al., 2012; Findilater et al., 2013).

However, despite the continuing growth of seniors using mobile reading devices and their great values to the elderly population, older adults typically show a strong preference for traditional printed books (Kretzschmar et al., 2013). Much research on the effectiveness and experience of different reading media has focused on the younger population, including students in primary and secondary school (Horton and Lovitt, 1994; MacCann et al., 2002; Sahin, 2011; Mangen et al., 2013; Porion et al., 2016; Wollscheid et al., 2016), and college (Schwartz et al., 1998; Ackerman and Lauterman, 2012; Stoop et al., 2013; Vincent, 2016) as new technologies continue to infiltrate in classrooms (Noyes and Garland, 2003; Mangen et al., 2013; Margolin et al., 2013; Millar and Schrier, 2015; Mizrachi, 2015; Aharony and Bar-Ilan, 2016). While the senior population has received some scholarly attention (Velikova et al., 1999; Kretzschmar et al., 2013; Fanning and McAuley, 2014), empirical research suggest that the elderly tend to lack confidence or have a certain degree of anxiety toward digital reading media as well as emerging technologies in general (Ellis and Allaire, 1999; Umemuro, 2004; Barnard et al., 2013). Such psychological factors could prevent seniors from reaping the myriad of benefits of using emerging technologies. To help older adults successfully use reading technologies and realize their potential benefits, it is important for researchers and technology designers to understand the specific users, their unique cognitive functions, psychological needs and preferences, and limitations (Di Giacomo et al., 2013). To this end, the current study aims to investigate how different reading media (screen versus paper) might result in different reading outcomes among older adults due to both cognitive and psychological factors.

As people age, they experience a number of cognitive changes that impair their reading performance. Recent studies (Li et al., 2013; Hou et al., 2017) suggested that a key factor that influences people’s reading performance lies in the extent to which the presentation of the reading text facilitates or impedes readers’ ability to form a cognitive map of the text. Similar to how people remember a physical landscape, such as the road taken to a place, readers form a mental map of the physical location of a text within a page. Both scholarly evidence and anecdotal experience testify that when people try to locate a particular piece of information they have read, they are often able to recall where in the text it appeared, such as a formula on the top of a right-hand page (Jabr, 2013). Thus, during reading, human brains not only gather information about a text but also process information about its context, and associates the two (Li et al., 2013; Hou et al., 2017). As such, individuals read by constructing a cognitive map of the text based on the spatial placement or presentation of the text on a page (Payne and Reader, 2006; Li et al., 2013; Hou et al., 2017).

Generally speaking, printed books have fixed layouts; such text presentation aids the formation of a physical map in readers’ minds of where certain pieces of information are. In contrast, screens typically make it difficult for readers to know the location of information in the document, thereby impeding readers in constructing an effective cognitive map. For example, scrolling text on a screen prevents readers from forming a coherent mental map, as there is no point for a reader to remember that a piece of text was on the top of page, because soon it might not be as the reader scrolls down the page (Piolat et al., 1997; Lee, 2005). The weak efficiency for forming cognitive maps impairs comprehension, and increases readers’ feeling of fatigue (Hou et al., 2017). Studies found that compared to young people, older individuals were less efficient in forming and using a cognitive map (Iaria et al., 2009). Difficulty with cognitive map processing might explain why older individuals particularly report difficulties with reading on screens (Iaria et al., 2009). To our knowledge, little research has investigated the influence of cognitive map formation on reading outcomes among older adults. We anticipate that a text presentation supporting the formation of a cognitive map of the text will bolster reading outcomes, while a text presentation with weak efficacy for forming a cognitive map will impair such outcomes for older adults.

Beyond the influence of cognitive modification in aging, psychological preferences of older adults with respect to technology could also play an important role. By tracking eye movements and brain activity of participants aged 60 and above as they read, a study suggested that older participants read faster and with less effort when reading from a tablet compared to a printed book. However, these participants still self-reported to gain more pleasure reading from a printed book and claimed that it was easier to read than reading on the tablet (Kretzschmar et al., 2013). These findings align with previous survey studies showing the strong subjective preference for reading on paper among older readers (Two Sides, 2015). The researchers suggested that the perception that digital devices reduce the pleasure of reading may be attributable to the positive attitude or cultural attachment associated with traditional printed books rather than a cognitive phenomenon (Kretzschmar et al., 2013). Thus, reading outcomes could potentially be affected by individuals’ psychological attitude toward the reading medium.

While psychological attitude toward technology varies across age groups, the psychological disposition of technophobia (Rosen and Weil, 1995a) is a particularly common and salient phenomenon among the senior population. Technophobia can be described as anxiety and overall negative attitudinal and affective response toward technology, its operation, or societal impact (Rosen and Weil, 1990). Technophobia could lead to physical symptoms, discomfort, and inefficiency (Brosnan, 2002; Gonzales and Wu, 2016). For example, an experimental study on technophobia revealed that individuals who experienced greater computer anxiety made more errors on a computer database searching task (Brosnan, 1998). Furthermore, longitudinal field studies have also demonstrated that technology anxiety can influence learning in technology-mediated environments (Fuller et al., 2006) and task achievement using technologies (Marcoulides, 1988). Research has documented evidence that individuals who have less past experience in using technologies are more likely to experience technophobia (Rosen and Weil, 1995b; Anderson, 1996). The elderly in general have significantly less technology experience than their younger counterparts (Dyck and Smither, 1994) whom many refer to as digital natives. With relatively less technological background, older people tend to lack confidence in their capabilities to understand and use technology, and as a result often feel insecure, discouraged, and stressed when using technology (Laguna and Babcock, 1997). As such, albeit having good access to technology, older adults showed a higher level of technophobia (Hogan, 2009) and exhibited lower performance in using technologies (Laguna and Babcock, 1997) compared to younger people.

## Materials and Methods

### Hypotheses

Taken together, existing literature implies that older adults may experience difficulties and frustration with technologies due to both cognitive and psychological influences. In this study, we aim to examine how reading on a screen versus paper might result in different reading outcomes among older adults due to both cognitive map and psychological attributes. As noted above, previous research (e.g., Li et al., 2013; Hou et al., 2017) suggested that the extent to which a text presentation facilitates or hinders the construction of a cognitive map of the text influences reading outcomes. Meanwhile, individuals’ psychological disposition of technophobia could also impact how they can effectively use technologies for reading. As such, we postulate that:

H1a. Individuals will display poorer comprehension performance when exposed to texts that have weak efficacy for forming a cognitive map.
H1b. Individuals will read slower when exposed to texts that have weak efficacy for forming a cognitive map.
H1c. Individuals will experience increased fatigue when exposed to texts that have weak efficacy for forming a cognitive map.
H1d. Individuals will experience increased general discomfort when exposed to texts that have weak efficacy for forming a cognitive map.
H1e. Individuals will rate experience as less enjoyable when exposed to texts that have weak efficacy for forming a cognitive map.
H2. Technophobia will be a significant covariate for the effect of cognitive map formation efficacy on reading comprehension (a), time (b), fatigue (c), general discomfort (d), and enjoyment (e).

While early research suggested that reading in print outperforms reading onscreen (e.g., Gould and Grischkowsky, 1984; Kurniawan and Zaphiris, 2001), recent studies found little evidence that people reading on paper show significantly different reading outcomes compared to people reading via digital technologies (e.g., Hou et al., 2017; Kretzschmar et al., 2013; Margolin et al., 2013). Due to the inconsistent findings, we tested the following non-directional hypotheses in the present study.

H3. Reading medium (paper vs. screen) will impact reading outcomes among older adults on reading comprehension (a), time (b), fatigue (c), general discomfort (d), and enjoyment (e).

### Experiment Design and Materials

This experiment employed a 2 (Medium: Paper vs. Screen) ^∗^ 2 (Cognitive Map Formation: Easy vs. Difficult) between-subject factorial design. The basic activities in the experiment involved reading texts and completing questionnaires. All experiment materials were in English. The reading material for this experiment was two texts of different types – a fictional story (i.e., a narrative text) and a scientific article (i.e., an expository text). Both were chosen from consumer publications intended for the general reading public, and were adapted by the researchers to be of similar length. The narrative text was 15-pages in length and had a total of 3,469 words. The expository text was also 15-pages in length and had a total of 3,150 words. None of the participants had read the texts prior to the experiment.

### Experiment Conditions

#### Reading Medium

The reading texts were presented on either a touchscreen tablet (Apple’s iPad) or on paper with identical font, font size, and line spacing. The tablet was 241 mm ^∗^ 186 mm ^∗^ 8.8 mm, with the display area being 193 mm ^∗^ 147 mm. The paper condition was the print version of the tablet condition. Thus, the two conditions had identical display size and page layout; the only difference was the reading medium.

#### Cognitive Map Formation

The text presentation was manipulated so that cognitive map formation of the text was either easy or difficult for participants. In the easy conditions, the reading texts were presented on a tablet screen or on paper in the format of a traditional book. In the screen version, participants swiped-through to turn pages on the tablet screen and saw one page at a time; this navigational method mimicked page turning similar to reading a physical book. In the paper version, participants were given a printed copy of the identical text used in the screen condition.

In the difficult conditions, scrolling was employed. Scrolling method has been shown to impede readers in forming cognitive maps because scrolling provides less contextual information during reading (Piolat et al., 1997; Lee, 2005). With paging many readers would get the contextual information where certain passage was on a page, such as whether it was at the left or right, top or bottom. With scrolling, however, pages are constantly changing as readers scroll through them. What is at the top at one second can be on the bottom the next; this weakens the association between text and pages, thus scrolling cannot effectively indicate a reader’s location in a text (Wästlund et al., 2008; Li et al., 2013). Therefore, we used scrolling as our manipulation for the difficult cognitive-map-formation conditions. In the screen version, participants read by scrolling texts upward to reveal succeeding pages. In the paper version, we employed a specifically designed paper scroll. Participants rolled the paper scroll upward as they read in a similar way as they scrolled on the tablet screen. The display size and page layout were held constant between the two reading media.

Additionally, across conditions, no additional reading activities, such as text highlighting, annotating, and bookmarking, were allowed. All participants were required to read in the portrait orientation.

### Participants and Procedure

The study obtained human subjects approval from the University Institutional Review Board. We obtained written informed consent from all participants, and they were compensated $10 for participating in the study.

#### Sample

A total of 81 older adults (*n* = 53 female, *n* = 28 male) aged 50 and above were recruited from a southeastern city in the United States and its surrounding areas. The participants ranged from 57 to 85 years of age with a mean age of 69.43 (*SD* = 6.01). They were randomly assigned to one of the four conditions with gender approximately balanced across the conditions.

#### Recruitment

Various methods were used for participant recruitment that included: social media, attendance at community meetings, interactions with agencies serving older adults (e.g., local senior services), and participant registries. A telephone screening was first conducted among potential participants to (1) describe the requirements of the study, and (2) to assess their eligibility status. Eligibility status was evaluated using the 10-item Short Portable Mental Status Questionnaire (SPMSQ; Pfeiffer, 1975). Participants making more than two errors on the SPMSQ may experience cognitive deficit that would prevent them from successfully participating in this study and bias our results, and thus were excluded. During our screening process, all participants passed the SPMSQ test.

#### Protocol

After being greeted to a quiet room on a university campus, participants were given an introduction to the experiment and finished the consent procedure. A pre-test paper-and-pencil questionnaire was administered first. Depending on their randomly assigned experimental condition, participants were asked to either read the two texts on a tablet or on paper. Prior to reading, all participants were given a demonstration by the experimenter on how to use their assigned reading medium. Participants were also instructed to read the texts at their normal pace, with the understanding that they would answer some comprehension questions afterwards. The experimenter recorded how long each participant took to finish reading, but participants were not made aware that they were being timed. After reading, participants completed the comprehension questions. The above steps were repeated with the second text. The order of the two reading texts was randomized. After reading the two texts, participants completed a post-test paper-and-pencil questionnaire. Finally, participants were debriefed and compensated. Most participants completed the experiment in approximately 50 min to 1 h.

### Measures

#### Comprehension

Participants were asked 20 comprehension questions, 10 questions for each text. One question was a sequence of events question; the rest were multiple-choice. On average, the number of correct answers ranged from 7 to 20, *M* = 15.730, *SD* = 2.579.

#### Time

The length of time it took participants to read was measured. This variable reflected participants’ reading speed. Participants completed the reading within 877–3087 seconds (i.e., 14.61–51.45 min), *M* = 1985.37 (i.e., 33.09 min), *SD* = 506.918.

#### Fatigue

Participants reported their feeling of fatigue while reading the texts on a 4-point scale (1 = *None*, 4 = *Severe*) in the post-test questionnaire, *M* = 1.120, *SD* = 0.399.

#### General Discomfort

Participants also reported their feeling of general discomfort (1 = *None*, 4 = *Severe*) in the post-test questionnaire, *M* = 1.280, *SD* = 0.617.

#### Enjoyment

Participants self-reported their reading experience on three items – Enjoyable, Fun, and Interesting, ranging from *Not at all* (1) to *Very much* (5). The items were averaged to create the scale score; higher scores on this scale denoted higher enjoyment of the reading experience, *M* = 3.551, *SD* = 0.881, Cronbach’s alpha = 0.82.

#### Technophobia

Technophobia was measured using five items adapted from the internet phobia scale from Brosnan and Rosen (2001). The five 7-point scale (1 = strongly disagree and 7 = strongly agree) items were: (1) “*I always feel anxious when using mobile devices*,” (2) “*I go out of my way to avoid using mobile devices and technologies*,” (3) “*It is easy for me to use mobile technologies* (reversed coded),” (4) “*My anxiety about mobile technologies bothers me*”, and (5) “*I am more anxious about mobile devices than I should be*.” The five items were averaged to create the technophobia scale, *M* = 2.094, *SD* = 1.099, Cronbach’s alpha = 0.84.

#### Experience in Mobile Technologies

Participants reported their experience with using mobile technologies on a 7-point scale. They were instructed to pick one of following choices regarding their mobile technology use: *Only once or twice in total* (1), *Less than once a month* (2), *A couple times a month* (3), *Once a week* (4), Several times a week (5), and *Every day* (6), or *Never use* (0). 54.3% (*n* = 44) of our sample reported using mobile technologies every day, while 21% of our sample reporting never having used mobile devices, *M* = 4.124, *SD* = 2.482.

#### Personality Characteristics (TIPI)

A brief measure of the Big-Five personality dimensions - the ten-item personality inventory (TIPI; Gosling et al., 2003) scale was used. The TIPI uses a 7-point Likert scale, and creates a score for each of the Big Five Personality traits: extraversion, openness to experiences, emotional stability, conscientiousness, and agreeableness.

## Results

### Statistical Analysis

Data analyses for this study were based on a series of full-factorial two-way ANCOVA using technophobia as a covariate. Technophobia was considered as an appropriate covariate that reflects the dispositional characteristic of individuals. As such, the two-way ANCOVA model was the fitted statistical test for the present study aiming to investigate the influences of both media factors (i.e., cognitive map formation and reading medium) and individual factor (i.e., technophobia) on older adults’ reading experience. Participants’ age, education level, and experience in mobile technologies were included in the analyses as control variables. In the *post hoc* test section, Pearson’s correlation tests were employed to further explore relationships between technophobia and other individual characteristics.

### Primary Results

Descriptive statistics for the study measures are presented in **Table 1**. The results of the ANCOVA analysis are shown in **Table 2**.

**Table 1:** Descriptive statistics of study variable by condition.

	Comprehension	Time (s)	Fatigue	Discomfort	Enjoyment
	*M* (*SD*)	*M* (*SD*)	*M* (*SD*)	*M* (*SD*)	*M* (*SD*)
Cognitive Map Formation: Easy (*n* = 40)	15.55 (2.61)	1988.90 (635.16)	1.03 (0.158)	1.33 (0.616)	3.46 (0.90)
Screen (*n* = 20)	15.00 (2.20)	2085.65 (737.33)	1.05 (0.224)	1.40 (0.754)	3.27 (0.95)
Paper (*n* = 20)	16.10 (2.91)	1892.15 (514.50)	1.01 (0.002)	1.25 (0.444)	3.65 (0.83)
Cognitive Map Formation: Difficult (*n* = 41)	15.90 (2.56)	2022.85 (531.00)	1.22 (0.525)	1.29 (0.784)	3.64 (0.86)
Screen (*n* = 21)	16.10 (2.57)	2076.24 (525.00)	1.14 (0.478)	1.20 (0.410)	3.57 (0.96)
Paper (*n* = 20)	15.70 (2.61)	1966.80 (544.98)	1.30 (0.571)	1.24 (0.624)	3.71 (0.75)
Total (*N* = 81)	15.73 (2.58)	2006.09 (581.33)	1.12 (0.399)	1.28 (0.617)	3.55 (0.88)

**Table 2:** ANCOVA analysis on cognitive map formation, reading medium, and technophobia.

Dependent Variables	Source of variation	Sum of squares	*df*	*MS*	*F*	η^2^
Comprehension						
	Cognitive Map Formation	4.577	1	4.577	0.750	0.010
	Reading Medium	1.397	1	1.397	0.229	0.003
	Technophobia	3.718	1	3.718	0.609	0.008
Time (seconds)						
	Cognitive Map Formation	207844.412	1	207844.412	0.940	0.013
	Reading Medium	163512.888	1	163512.888	0.740	0.010
	Technophobia	1382942.071	1	1382942.071	6.256^∗∗^	0.079
Fatigue						
	Cognitive Map Formation	1.039	1	1.039	6.975^∗∗^	0.087
	Reading Medium	0.082	1	0.082	0.552	0.008
	Technophobia	0.513	1	0.513	3.440^∗^	0.045
Discomfort						
	Cognitive Map Formation	0.013	1	0.013	0.036	0.000
	Reading Medium	0.233	1	0.233	0.641	0.009
	Technophobia	2.328	1	2.328	6.394^∗∗^	0.081
Enjoyment						
	Cognitive Map Formation	0.338	1	0.338	0.448	0.006
	Reading Medium	0.943	1	943	1.25	0.017
	Technophobia	0.009	1	0.009	0.012	0.000

*^∗∗^p < 0.05, ^∗^p < 0.10.*

H1 expected that when exposed to texts that have weak efficacy for forming a cognitive map, individuals would show poorer reading outcomes, including displaying poorer comprehension performance (a), taking more time to read (b), experiencing greater feeling of fatigue (c) and general discomfort (d), and enjoying the reading less (e).

The ANCOVA analyses testing H1a and H1b failed to detect significant effects of cognitive map formation on comprehension and reading speed, respectively. However, the analyses indicated a significant effect of education on reading comprehension, *F*(1,73) = 6.07, *p* = 0.016, η^2^ = 0.08. In testing H1b, a significant effect of education on reading speed was also found, *F*(1,73) = 11.384, *p* = 0.001, η^2^ = 0.135. The results suggest that seniors with higher levels of education scored better on the comprehension questions and spent less time reading the texts. But H1a and H1b were not supported.

Consistent with H1c, the results of the ANCOVA revealed a significant main effect of cognitive map formation on fatigue, *F*(1,73) = 6.975, *p* = 0.010, η^2^ = 0.087. Participants in the scrolling conditions reported stronger feelings of fatigue than those in the paging conditions. H1c was supported. There was no significant main effect for cognitive map formation on feelings of general discomfort or enjoyment of reading. H1d and H1e were not supported.

It was also hypothesized (H2a–e) that technophobia, measured as an individual characteristic in the pre-test, would significantly influence the reading outcomes. The ANCOVA analyses showed that technophobia had a significant effect as a covariate for reading speed, *F*(1,73) = 6.256, *p* = 0.015, η^2^ = 0.079, and for general discomfort, *F*(1,73) = 6.394, *p* = 0.014, η^2^ = 0.081. Participants who had a higher level of technophobia spent more time reading the texts and showed stronger feelings of general discomfort while reading. Technophobia also had a marginally significant effect on fatigue, *F*(1, 73) = 3.440, *p* = 0.068, η^2^ = 0.045. That is, participants with a higher level of technophobia also reported increased feelings of fatigue compared to those with a lower level of technophobia. Thus, both H2b and H2d were supported. H2c was supported with marginal significance. H2a and H2e were not supported.

H3 postulated that reading medium (paper vs. screen) would impact reading outcomes among older adults on reading comprehension (a), time (b), fatigue (c), general discomfort (d), and enjoyment (e). The ANCOVA analyses, however, did not show significant differences between the conditions when older adults read on digital screens as opposed to paper in any of the five reading outcomes. Thus, H3a–H3e were not supported.

### *Post hoc* Analyses

To further understand the psychological disposition of technophobia and, in particular, to explore who (in terms of demographics and personality traits) is more likely to have a high level of technophobia, Pearson’s correlational analyses were conducted to examine associations between technophobia and individual variables.

Our data showed that technophobia was negatively correlated with openness to experiences, *r* = -0.355, *p* < 0.001, agreeableness, *r* = -0.266, *p* = 0.017, and emotional stability, *r* = -0.233, *p* = 0.037. Technophobia was also negatively correlated with experience in mobile technologies, *r* = -0.521, *p* < 0.001. It also showed a marginally significant relationship with age, *r* = 2.12, *p* = 0.058 (two-tailed). Technophobia was not correlated with education level, and it did not differ between male (*M* = 2.30, *SD* = 1.25) and female (*M* = 1.98, *SD* = 1.01), *t*(79) = -1.275, *p* = 0.206. **Table 3** presents the results of the correlation tests.

**Table 3:** ANCOVA analysis on cognitive map formation, reading medium, and technophobia.

Variable	1	2	3	4	5	6	7	8	9
1. Technophobia	1								
2. Openness to experiences	-0.355**	1							
3. Agreeableness	-0.266*	0.128	1						
4. Emotional stability	-0.233*	0.145	0.267*	1					
5. Conscientiousness	-0.120	0.055	0.036	0.292**	1				
6. Extraversion	-0.168	0.344**	0.090	0.093	0.104	1			
7. Technology use	-0.521**	0.275*	0.138	0.284*	0.229*	0.108	1		
8. Age	0.212	-0.267*	-0.039	0.189	0.056	-0.065	–0.175	1	
9. Education	-0.014	0.014	-0.027	-0.209	-0.128	-0.146	–0.058	–0.020	1

*Two-tailed ^∗∗^p < 0.001, ^∗^p < 0.05.*

## Discussion

The purpose of this study was to investigate the effects of cognitive map formation and reading medium on reading performance and experience among older people in terms of comprehension, reading speed, fatigue, general discomfort, and enjoyment. It also investigated the influence of technophobia on these reading outcomes. Our results showed that the efficacy of cognitive map construction influences readers’ feeling of fatigue. This finding is in line with existing literature (Hou et al., 2017) suggesting that reading a text that hinders cognitive map formation is more tiring for readers because they need to constantly adjust the processing of the text in relation to its spatial placement on the page. Our results replicated existing findings and extended it to the elderly readers. However, we failed to observe compromised comprehension performance in the conditions that was difficult for readers to form an effective cognitive map, a finding that has been documented in previous studies (e.g., Li et al., 2013; Margolin et al., 2013; Hou et al., 2017). It is possible that general participants in our sample achieved high comprehension accuracy. In the experiment, we allowed sufficient time for participants to read, and participants were made aware that they would be tested with comprehension questions after reading. This might have motivated them to read the texts carefully to prepare for the test. Future studies could consider allocating a time limit to the reading task, or measure comprehension while controlling the influence of motivation. Another possible explanation is that since comprehension was strongly influenced by readers’ education level, as was shown in our analysis, and that our participants had overall high education level, most participants were able to do well on the comprehension questions. In sum, we suggest it requires further investigation in future research to see how the cognitive map mechanism influences comprehension among older adults. Furthermore, in line with previous studies (Kretzschmar et al., 2013; Hou et al., 2017), our data did not show significant differences between reading on screen versus on paper in terms of reading performance and experience. Thus, we add another piece of empirical evidence to the recent postulation that reading on digital media could match that of reading from paper if the representation of the document digital reading devices resembles that of the print book (Hou et al., 2017).

Our findings highlight the importance of a psychological disposition factor – technophobia – that could threaten older adults’ ability to effectively use digital technologies for reading. In the current study, older adults with higher levels of technophobia spent more time on reading, but they performed as well on reading comprehension as those with lower levels of technophobia. This finding was consistent with Darke’s (1988) suggestion that anxious individuals allocate more cognitive resources to off-task efforts such as worrying about their performance, and as a result are slower to complete the actual tasks, but do not necessarily make more errors. Our finding resonated with previous research showing that the people who experience more computer-associated anxiety may exhibit lower self-efficacy to perform reading well on digital media (Brosnan, 1998), thereby showing slower reading speed.

It is worth noting that previous studies have mainly focused on the negative influence of technophobia on performance of a novel computer-based task, such as database searching (Brosnan, 1998), data entry (Mahar et al., 1997), and word-processer usage (Brosnan, 1999), which require some level of computer literacy and software operating skills. Thus, one can argue that the negative impact of technophobia on performance might stem from individuals’ reluctance to learn a novel skill. Few studies, however, have looked at daily actives that are mediated by computers, yet require minimum learning of novel skills, such as reading on a touch-screen mobile device. Reading on a touch-screen mobile device is not a computer-based task in the traditional sense because the intuitive user interface of the reading devices nowadays requires little effort from the users to perform any computer operations. Therefore, the negative impact of technophobia on reading performance is likely to be a result due to users’ negative attitudes directly towards computer itself rather than negative attitudes towards computer-based operations. Meanwhile, reading on a touch-screen is becoming increasingly incorporated into our everyday experience, which may have important consequences for people, especially older adults, with technophobia. In testing the effects of older adults’ technophobia on reading on a touch-screen, we contribute to previous technophobia literature by analyzing a new yet common context of technology use, and by further clarifying if technophobia is a result of the negative attitudes towards the computer itself or the idea of having to perform computing operations.

Interestingly, our results also showed that older adult users with higher level of technophobia experienced more subjective feeling of discomfort and fatigue. This finding suggested that despite that older adult users performed minimum computer operations during the reading, just the thought of reading on a computer device would activate negative responses within users who possessed pre-existing negative posture toward computers. On a different research topic, an experimental study by Gonzales and Wu (2016) found a similar pattern among college students with higher level of technophobia. When excluded by someone reading on a cellphone in their presence, college students who harbor more negative attitudes toward computer technology reported worsened mood, a reduced sense of self-esteem and control over their environment. Our study results extend these findings, and contribute to the broad research dialog regarding how technophobia may deter the process of human-computer interaction.

Moreover, our *post hoc* analyses explored the associations between technophobia and individual characteristics. Overall our results were consistent with past research examining technophobia. For instance, existing research indicated that previous computer experience and technology ownership is a main predictor of technophobia (e.g., Maurer, 1994; Chua et al., 1999). Our data also showed that as an individual’s experience in technology use increases, their level of technophobia decreases. Further, studying older adults between the ages of 60 to 97, Ellis and Allaire (1999) found that age was positively correlated with computer anxiety. Similarly, our data indicated a marginally significant relationship between age and technophobia. Furthermore, we provided some indications that technophobia could be associated with an individual’s personality traits (Anthony et al., 2000), particularly one’s openness to experience. As a person is more open to innovative experiences, s/he is less likely to experience technophobia. Future studies could employ other research methods, such as large-scale survey or in-depth interview, to further understand what may cause technophobia and how older adults can overcome it.

Unlike the youth population, growing up as digital natives, who are more likely to face problems such as internet addiction, the senior population might have an opposite concern associated with anti-digital-reading-attitudes and anxiety in using technologies. Thus, research-driven evidence-based technology design and interventional programs that understand this population group and accommodates for their unique cognitive functions and psychological needs could lead to development of innovative technologies that bring important benefits to the senior population, and in turn, promoting successful aging as a whole. This issue becomes increasingly important as the global aging population continues to grow and individuals’ life expectancy gets longer. In sum, while we embrace a variety of positive impacts of using technology on seniors’ lives (Topo, 2009), the degree to which the technology innovation is compatible with one’s values, experiences, and needs (Rogers, 2003) should receive more scholarly attention.

### Limitations and Future Research

This study has limitations that warrant discussion and can inform future studies. First, self-selection bias is considered a limitation to our study. It was likely that older people who expressed interest in participating in our study were more techno-savvy than the general aging population, because many of our participants were able to respond to the study advertisements sent via email and on social media. Second, due to the voluntary nature of subject recruitment, the composition of our sample was primarily Caucasian with a relatively high education level on average. Previous research has revealed that individuals’ demographic characteristics, such as ethnicity and socioeconomic status are associated with technophobia (e.g., Rosen and Weil, 1995b). Hence, this study sample could have potentially excluded a technologically underserved population who experiences higher level of technophobia. Future studies should pay attention to the sample diversity in order to make more generalizable conclusions regarding digital reading. Lastly, our measure of fatigue and general discomfort relies on a single self-reported item. Future research could employ more comprehensive self-report measures or more objective measures targeting the older adult population to triangulate our findings.

## Conclusion

Our study provides implications for scholars, technology designers, and training program practitioners to promote digital reading among aging users. While existing literature and interventional programs mainly focus on improving access to digital devices and increasing digital literacy among seniors, our study further highlights the importance of providing tailored support that helps overcome psychological obstacles in using technologies for the aging population.

## Ethics Statement

This study was carried out in accordance with the recommendations and approval of “Thomas L. Jacobson” from the Human Subjects Committee of the involved Institute with written informed consent from all subjects. All subjects gave written informed consent in accordance with the Declaration of Helsinki.

## Author Contributions

This study was led by JH: she conceptualized and designed the study, ran experiments, collected data, performed the data analysis, and wrote the paper. YW filed the IRB application, recruited participants, ran experiments, collected data, and contributed to the manuscript write-up. EH recruited participants, and collaborated to data collection. All authors edited the paper and approved the final manuscript. JH takes primary responsibility for the research reported here.

## Conflict of Interest Statement

The authors declare that the research was conducted in the absence of any commercial or financial relationships that could be construed as a potential conflict of interest.
